# Can Diversifying Selection Be Distinguished from History in Geographic Clines? A Population Genomic Study of Killifish (*Fundulus heteroclitus*)

**DOI:** 10.1371/journal.pone.0045138

**Published:** 2012-09-26

**Authors:** Allan E. Strand, Larissa M. Williams, Marjorie F. Oleksiak, Erik E. Sotka

**Affiliations:** 1 College of Charleston, Department of Biology, Grice Marine Laboratory, Charleston, South Carolina, United States of America; 2 Woods Hole Oceanographic Institution, Department of Biology, Woods Hole, Massachusetts, United States of America; 3 Rosenstiel School of Marine and Atmospheric Sciences, University of Miami, Miami, Florida, United States of America; University of Arkansas, United States of America

## Abstract

A common geographical pattern of genetic variation is the one-dimensional cline. Clines may be maintained by diversifying selection across a geographical gradient but can also reflect historical processes such as allopatry followed by secondary contact. To identify loci that may be undergoing diversifying selection, we examined the distribution of geographical variation patterns across the range of the killifish (*Fundulus heteroclitus*) in 310 loci, including microsatellites, allozymes, and single nucleotide polymorphisms. We employed two approaches to detect loci under strong diversifying selection. First, we developed an automated method to identify clinal variation on a per-locus basis and examined the distribution of clines to detect those that exhibited signifcantly steeper slopes. Second, we employed a classic 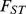-outlier method as a complementary approach. We also assessed performance of these techniques using simulations. Overall, latitudinal clines were detected in nearly half of all loci genotyped (i.e., all eight microsatellite loci, 12 of 16 allozyme loci and 44% of the 285 SNPs). With the exception of few outlier loci (notably mtDNA and malate dehydrogenase), the positions and slopes of *Fundulus* clines were statistically indistinguishable. The high frequency of latitudinal clines across the genome indicates that secondary contact plays a central role in the historical demography of this species. Our simulation results indicate that accurately detecting diversifying selection using genome scans is extremely difficult in species with a strong signal of secondary contact; neutral evolution under this history produces clines as steep as those expected under selection. Based on these results, we propose that demographic history can explain all clinal patterns observed in *F. heteroclitus* without invoking natural selection to either establish or maintain the pattern we observe today.

## Introduction

Clinal variation is a common feature of terrestrial, freshwater [1], [2] and marine species [3]–[5]. This list includes *Homo sapiens*, whose geographic patterns of polymorphism are largely clinal [6]. In a review of John Endler’s influential monograph on geographic clines, David Woodruff quipped that clines and hybrid zones are “taxonomist’s nightmares and evolutionist’s delights” because they inform several vexing issues in evolution, including natural selection, gene flow, historical demography, and the definition and generation of species [7], [8].

Historically, clinal patterns were considered to be the consequence of diversifying selection [9], especially in cases in which a cline coincides with geographic shifts in abiotic or biotic factors (or an ecotone [7]). It is now broadly recognized that diversifying selection is not a necessary condition for establishment of clinal patterns. Restricted dispersal can create seemingly adaptive clines at single loci [10], and historic geographic subdivision may establish clinal patterns that may persist for long periods of time [7], [11] especially when population density or gene flow is low [12]. Despite a long history of both theoretical and empirical investigation into clinal variation, the extent to which selection, gene flow, history, and their interactions contribute to shaping a particular cline remains a difficult, and largely unanswered question. Today, the challenge that remains is to understand the extent by which particular clinal patterns are due to these neutral and selective processes [5]. For a well-developed theory of clines to successfully explain these commonly observed patterns, it will be critically important to distinguish the role of selection from other evolutionary factors.

With the recent explosion of genomic techniques to perform genome scans, evolutionary geneticists working with non-model organisms find themselves on the cusp of discovering loci under diversifying selection at relatively modest cost [13], [14]. Arguably, however, advances in the analysis of genomic data lags advances in the generation of genomic data, and this limits our ability to fully capitalize on the genomic information. One of these disparities is well illustrated by genomic clinal patterns.

One of the most successful approaches to identifying candidate loci under diversifying selection is the 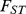-outlier technique [15], [16], which requires measurement of geographic differentiation (typically measured as F*_ST_*) across two or more populations at multiple loci. Loci under strong diversifying selection are those whose F*_ST_* values are larger than expected from coalescent simulation of neutral evolution in an island model [15]. In the case of strong clines, however, both ‘neutral’ and ‘non-neutral’ loci show similar patterns of geographic variation [5], and 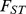 values between edge populations are often uniformly high for all loci. As a consequence, it is challenging to distinguish among neutral and non-neutral patterns when also accounting for underlying clinal patterns. Simply ignoring the underlying clinal pattern may dramatically increase the rate of false identification of loci under diversifying selection. An additional limitation of 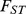 analyses in clines is that they ignore potentially useful information encoded in characteristics of the cline such as its location and slope [17].

One of the most widely-cited and well-characterized genetic clines occur within the killifish *Fundulus heteroclitus*, a common estuarine fish whose biology and ecology has been intensively studied for over a century[18]–[20]. Over this period, *F. heteroclitus* has been developed as an economically important species, constituting a significant proportion of bait sold in commercial fish bait operations [21]. Perhaps more importantly, *F. heteroclitus* has been developed as a model system for experimental biology [18], and especially as a model for environmental toxicology in estuarine systems [20].

**Figure 1:**
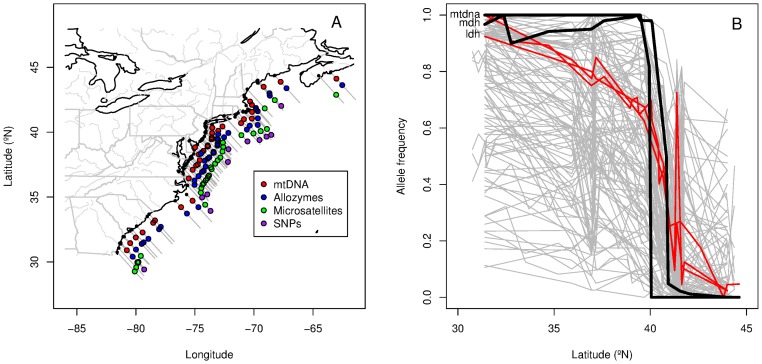
Sampling locations and allele frequencies in *Fundulus heteroclitus*. A) Location of sampled populations. B) Allele frequences of clinal loci used in the Genomic Co-Co plot analysis. mtDNA and Mdh allozyme clines are highlighted in black, and Ldh allozyme and Ldh SNP clines are highlighted in red.

*Fundulus heteroclitus* also became an important model organism for the study of adaptive clinal variation [22]–[24] for several reasons. There are coincident allozyme clines centered at approximately 40°N along the New Jersey shoreline [25]–[27] (figure 1). These allozyme clines correlate with latitudinal changes in air and water temperature: the average yearly water temperature decreases by approximately 1°C per degree latitude [25]. A number of functional studies, especially those examining lactate dehydrogenase (Ldh), have indicated that protein variants (at the enzymatic, cellular and organismal levels) differ in their performance across these temperatures [25], [28], [29], suggesting that selection plays a role in maintaining *F. heteroclitus* allozyme clines. At the same time, non-adaptive explanations for *Fundulus* clines have strong empirical support. Most putatively-neutral loci that have been examined (mitochondria [30]–[32], microsatellites [23], [33] and SNPs [34]) display clinal variation that mirrors that of many allozyme loci (figure 1). Moreover, at microsatellite and mitochondrial SNPs, there is substantial endemic diversity at both northern and southern ends of the cline [35]. These patterns are consistent with a secondary contact zone between historically distinct populations and suggest that neutral non-equilibrial processes may play a role in generating and maintaining *F. heteroclitus* clines. While authors have consistently recognized that allozyme clines may reflect neutral processes in *F. heteroclitus* [19], [23], [25], [32], a comprehensive, simultaneous analysis of all publically available allelic clines has yet to be pursued. Such an analysis has the potential to discern the degree to which putative loci are under selection or reflect neutral processes (gene flow, drift, non-equilibrial secondary introgression).

Here, we apply two general analytical approaches to detect the differences in spatial distributions of *F. heteroclitus* alleles undergoing diversifying selection and those evolving neutrally. One approach is the 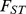-outlier technique [15], [16]. The second approach focuses upon landscape-level parameters of the clines themselves, in particular, their location and slope. Szymura and Barton [12] pioneered the use of distributions of cline locations and slopes at multiple loci to simultaneously examine both demographic and selection history contained in a cline between two species of fire-bellied toads. In this study, we implement an analysis to visualize the distribution of cline location (**co**incidence) and slope (**co**ncordance) for large numbers of loci. We call the resulting plot a genomic Co-Co plot. An analogous approach has been used successfully to show that clines in morphological traits had different midpoints [36] and steeper slopes [37] than did the ‘neutral’ expectation, suggesting these morphological traits were under selection. Finally, we simulate population genetics of neutral loci under a likely demographic history for *F. heteroclitus*. We then use the simulated genotypic distributions to examine the performance of both 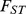 outlier and Co-Co analyses in the presence of secondary contact. Our results caution that inferring selection using genome scans is very difficult when a strong signal of secondary contact remains.

## Results

### Summary of Genetic Data

We assembled a combined dataset in *F. heteroclitus* that consists of 310 loci (1 mtDNA, 8 microsatellites, 16 allozymes, and 285 SNPs) from the sources available listed in Table 1. Not all loci were sampled at each location; Figure 1a indicates the spatial locations for loci from each category (mtDNA, allozyme, microsatellite, and SNP). Close examination of Figure 1a reveals fine-scaled spatial sampling for allozymes and mtDNA, and slightly less dense sampling for microsatellites. Though the majority of the loci examined in this study were of SNP origin, these data were the least densely sampled with a total of 11 populations included, though the spatial extent of sampling for SNPs did span the cline located at approximately 40°N.

**Table 1:** Sources and types of data employed in this study.

Study	Genetic loci
Powers and Place (1978) [25]	*Pgm-A* (phosphoglucomutase-A)
	*Gpi-B* (glucosephosphate isomerase-B)
	*Mdh-A* (malate dehydrogenase-A)
	*Ldh-B* (lactate dehydrogenase-B)
Cashon et al. (1981) [26]	*Est-S* (serum esterase)
	*6-Pgdh-A* (6-phosphogluconatedehydrogenase)
	*Idh-A* (isocitrate dehydrogenase-A)
	*Idh-B* (isocitrate dehydrogenase-B)
Ropson et al. (1990) [27]	*Est-b* (liver esterase-B)
	*Ap-A* (acid phosphatase-A)
	*H6pdh-A* (hexose-6-phosphatedehydrogenase-A)
	*Pgm-B* (phosphoglucomutase-B)
	*Fum-A* (fumarase-A)
	*Mpi-A* (mannosephosphate isomerase-A)
	*Aat-A* (asparate aminotransferase-A)
	*Aat-B* (asparate aminotransferase-B)
Smith et al. (1998) [31]	RFLP-mtDNA
Duvernell et al. (2008) [23]	8 microsatellite loci
Williams et al. (2010) [39]	336 SNP loci (only 285 used)

### Comparison among Types of Loci

Of the 310 loci used in this study, 146 (47%) exhibited a clinal pattern of variation based upon likelihood ratio tests between intercept only models and broken-stick models assessed at the 0.05 level. In the SNP dataset alone, 44% of the loci genotyped in *F. heteroclitus* exhibit a detectable clinal pattern (Table 2).

**Table 2:** Joint distribution of locus type and presence of clinal pattern for at least one allele based on broken-stick fits.

Type of Locus	Clinal Pattern	No Clinal Pattern
Allozyme	12	4
mtDNA	1	0
Microsatellite	8	0
SNP	125	160

Table 2 also illustrates the difference in the distribution of clinal patterns of variation among marker types. While the mtDNA locus and the majority of microsatellite and allozyme loci exhibit clinal variation, slightly less than one-half of SNPs did. This difference in clinal frequency between SNP and non-SNP loci is strongly supported by contingency-table analysis (Fisher’s exact test, p = 0.0001).

### Outlier Analyses

**Figure 2:**
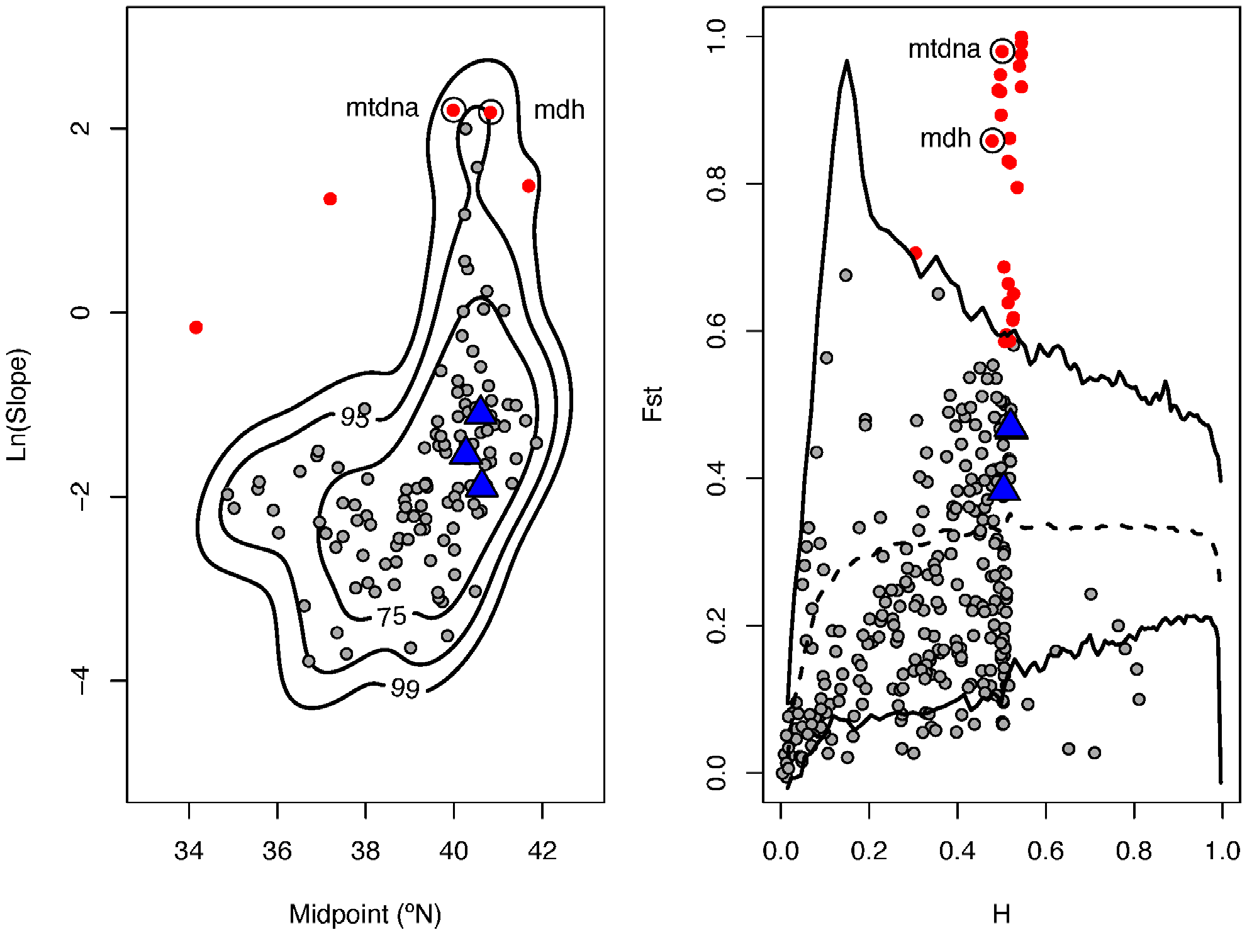
Identification of outlier loci based on Genomic Co-Co plot (left panel) and fdist2 (right panel) analyses. Contours of 75, 95 and 99% are shown on the co-co plot. Mean F*_ST_* is dotted and 99% C.I. lines are in black on the fdist2 panel. Outlier loci are in red (95% C.I.), non-outlier loci are in grey, and Ldh allozyme and SNPs are blue.

The Co-Co plot indicates that the average midpoint of clinally-distributed loci is centered at approximately 40°N, with a broad confidence interval at both midpoint and slope (Figure 2, left panel). Outlier loci in the Co-Co plot tend to have a steeper slope and most have a midpoint that is 40°N. The fdist2 analysis (Figure 2, right panel) indicates that over most values of heterozygosity, F*_ST_* values average 0.3. This reflects the high degree of differentiation across *F. heteroclitus* noted previously (see Introduction). In our fdist2 analysis, outlier loci are those that have a more profound differentiation than expected. No loci with a weaker level of differentiation were significantly distinct, but this is not unexpected given that fdist2 is known to provide greater statistical power in detecting divergent relative to stabilizing selection [15].

Two loci (mtDNA and malate dehydrogenase, or Mdh) were significant outliers at both F*_ST_*-outlier and Co-Co plot analyses. Twenty five SNP loci were significant outliers at only F*_ST_*-outlier and three SNPs were significant outliers with only Co-Co plot analyses (Table S1).

Neither F*_ST_* outlier nor Co-Co analyses indicate that alleles at Ldh are under diversifying selection. In fact, both analyses place Ldh in the center of the joint distributions of 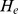 and F*_ST_* in the F*_ST_* outlier analysis and cline slope and midpoint in the Co-Co analysis (Figure 2, Figure 1B). This pattern is consistent among Ldh genotypes derived from allozyme analysis as well as genotyping based on two SNPs found within Ldh [38]. The fact that both allozyme and SNP characterization of Ldh genotypes show the same pattern, while anecdotal, also supports our decision to combine datasets to develop the large number of markers needed to identify outlier loci.

### Simulations

**Figure 3:**
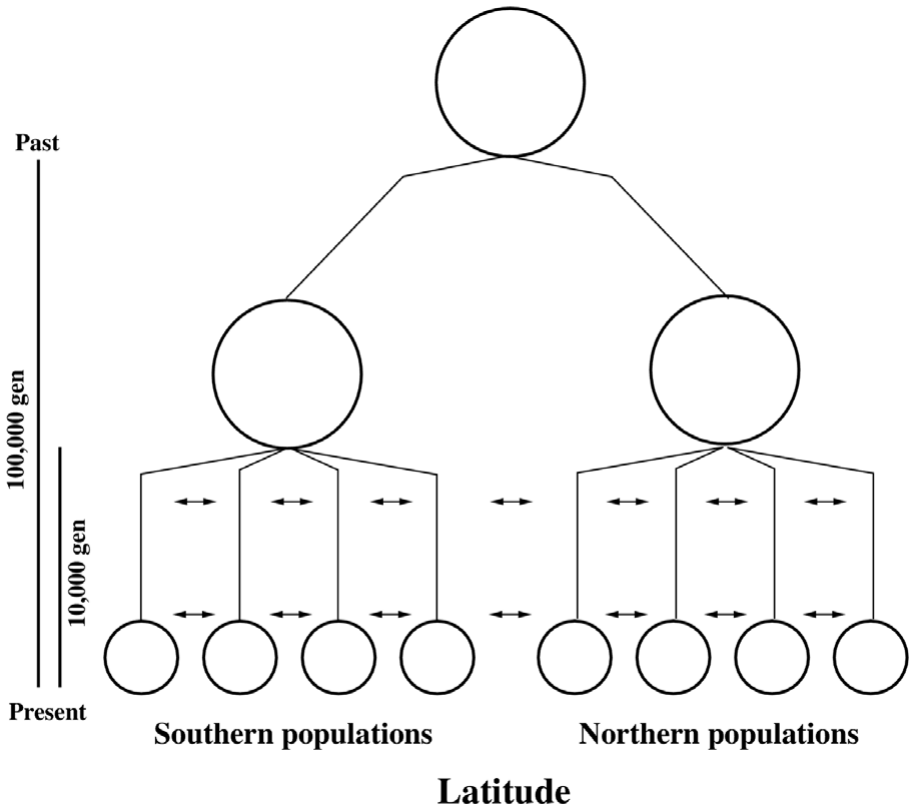
Demographic model for simulation study. For all simulations presented, ancestral populations are split into two relictual populations 100,000 generations in the past. At 10,000 generations before present, each of these daughter populations split into 20 linearly arranged populations (only 8 of the resulting 40 populations are indicated). Gene flow between populations continued at the same rate throughout the 10,000 generations.

**Figure 4:**
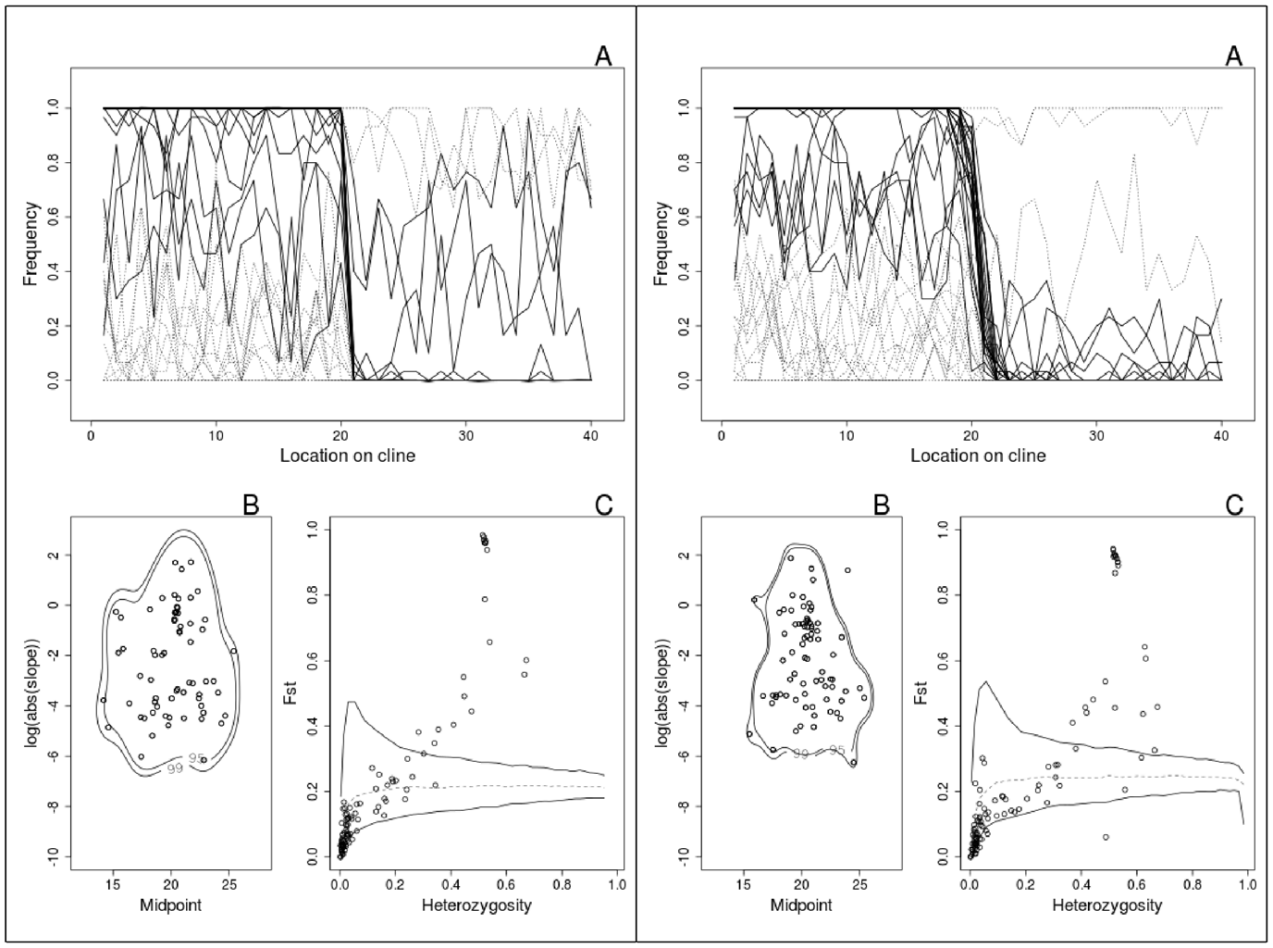
Summaries of two simulations. In the sub-figure on the left the stepping-stone dispersal rate is 0.4 (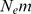); on the right, 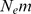 is equal to 4. In each sub-figure, panel **A** illustrates the distribution of allele frequencies along the cline for simulated loci. All clines are adjusted so that higher allele frequencies are on the left. Bold lines indicate loci deemed to be under diversifying selection by fdist2. Panel **B** shows the distribution of cline midpoints and slopes estimated using broken stick models applied to the clines in panel **A**. Panel **C** shows the results of fdist2 applied to these clines.

To assess the performance of both outlier approaches to detect loci under diversifying selection along clines, we simulated neutral loci in the *Fundulus* system along the western North Atlantic. Figure 3 outlines the design that we used for these simulations. Figure 4 summarizes simulation results under two different rates of dispersal (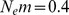 and 4.0, respectively). Our simulations indicate that after 10K years since the introgression of historically-separated populations of *Fundulus*, a large proportion (42%) of neutrally-simulated loci maintained a clinal pattern. Dispersal had a small but discernible effect upon cline width (Figure 4; for higher dispersal rates, see Figure S2 in supporting information). In addition, the percent of loci exhibiting a clinal pattern increased somewhat with dispersal rate (mean percent of clinal loci are 36%, 41%, and 50% for 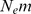 equal to 0.4, 4, and 40, respectively). As expected under neutrality, equal numbers of clines exhibited positive and negative slopes (in Figure 4 A-panels, 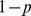 was plotted for clines with positive slopes).

Subjecting simulation results to the two outlier analyses yielded overall distributions of cline steepness and among population 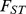 largely indistinguishable from the same analyses performed upon the empirical data (Figure 2 vs. Figure 4). As we observed in the empirical data, large numbers of neutrally simulated loci were identified as undergoing diversifying selection. In addition, the peak in 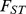 values identified at intermediate heterozygosities in the empirical data were also present in the simulated datasets, regardless of the strength of gene flow.

## Discussion

To our knowledge, this study represents the first time that the relative contribution of neutral and non-neutral processes has been quantified in the classic genetic clines of the killifish *Fundulus heteroclitus*. In addition, our computer-based coalescent simulations replicated the general characteristics of empirical *F. heteroclitus* clines in the absence of selection. This result raises real concern about the power of SNP genome scans to detect ‘loci that matter’ when a large portion of clines across the genome are generated by secondary contact.

### Clinal Frequencies among SNPs versus Other Loci

Fewer SNP loci show clinal variation than observed in the other marker types (44% versus 75–100%; Table 2). Barring a fundamental difference in the evolutionary dynamics of SNPs versus other types of marker loci, this result could reflect bias associated with polymorphism screening, variability in numbers of segregating alleles per locus, or both. In the former, it is likely that published microsatellite and allozyme loci were chosen non-randomly from a larger pool of loci based upon their pattern of geographic variation. This easily could have occurred if new markers were screened for polymorphism by comparing few individuals collected from the margins of the range of *F. heteroclitus*. Using this common approach for assessing utility of genetic markers, loci that appear polymorphic differ in allele frequency at the ends of the cline, a necessary condition for clinal variation. SNP loci on the other hand, were chosen from the *F. heteroclitus* genome based upon location of restriction endonuclease recognition sites [39] and were not subsequently filtered based on patterns of geographic variability prior to this study. Alternatively, the typically bi-allelic nature of SNP loci results in a situation where the pattern of geographic variation for one allele is the complement of the other. In multi-allelic marker systems like allozymes, and in particular microsatellites, there are *k−1* distinct geographic patterns possible among the alleles segregating across the species range, where k is the number of alleles segregating at a locus. Therefore when considering geographic variation on a per-locus basis as we have done here, mutli-allelic loci have more chances to exhibit a clinal pattern, even if some alleles at that loci are distributed non-clinally.

### Simulations of *F. heteroclitus* Demographic History

Simulating the likely history of *F. heteroclitus* populations and analyzing simulation output with our approach yielded patterns that were remarkably similar to those observed in the empirical dataset (Figure 2 vs. Figure 4, B and C panels). Furthermore, the pattern revealed in the empirical data by the Co-Co and fdist2 analyses persisted under different rates of gene flow (Figure 4 left and right panels; Figure S2).

The proportion of loci that exhibited clinal patterns in the simulations bracketed the proportion observed in SNPs and in the entire genetic dataset (44% and 47%, respectively; Table 2). The difference depended on dispersal rate. At the lower rates of dispersal simulated in Figure 4, the percent of neutral loci exhibiting a clinal pattern was smaller than observed in the entire empirical dataset (one sample t-test: 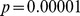 and 0.0001 for 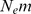 equal to 0.4 and 4, respectively). At higher dispersal rates, this proportion of clinal loci was higher than observed overall (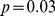 for 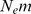 equal to 40).

It is clear from both empirical and simulated fdist2 and Co-Co analyses that no signal of diversifying selection could be teased away from the strong demographic history signal in this system, despite the initially suggestive large numbers of outliers in these analyses. An interesting artifact associated with the fdist2 approach emerges from both empirical and simulated datasets and suggests the coalescent simulation model employed by fdist2 may always be problematic in clines with a history of secondary contact. A distinctive peak in 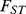 values occurs at a system-wide 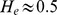. This peak occurs at an 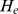 that results when each end of the cline is fixed for alternative alleles. This is the pattern exhibited by mtDNA and MDH in the empirical dataset. In this situation, the majority of populations are fixed for one allele or the other depending on their location along the linear cline resulting in almost all genetic variability occurring among populations (high 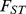). This artifact was predicted by Beaumont and Nichols [15] when they examined the performance of fdist under different demographic scenarios and observed that clusters of similar populations effectively reduced the number of populations providing information. In the extreme case, strong linear clines represent two clusters of populations. Other authors have noted this issue as well under ancient vicariance followed by local range expansion [40]. The population-level history of *F. heteroclitus* through the Pleistocene and Holocene exemplifies this history [35].

### Independent Evidence of Selection at *F. heteroclitus* Loci

#### mtDNA

Both analyses find that mitochondrial RFLP haplotypes have a significantly steep clinal slope. Moreover, fdist2 identified a mitochondrial SNP (Table S1) that had significantly greater F*_ST_* than expected. These results weakly support the hypothesis that maintenance of the mitochondrial cline since secondary contact may result from diversifying selection. Independently, environmentally-mediated selection has been proposed previously for mitochondria [32], [41]. It is also possible that cytonuclear co-adaptation may minimize the introgression of mitochondrial alleles across the cline [42]–[44], but this remains to be tested in *Fundulus*.

#### MDH

Both F*_ST_*-outlier and Co-Co plot analyses consistently highlight the significantly steeper cline at cytosolic malate dehydrogenase (or Mdh). It was previously recognized that Mdh “exhibits a substantially steeper cline” than the better understood Ldh, and ‘that selection may be even stronger at the locus’ [19]. However, ours represents the first effort to assess this uniquely strong cline among a large sample of coincident clines. Very little functional work on Mdh isozymes has been published for *Fundulus* even while Mdh is known to mediate temperature tolerance among other marine organisms [45]. Furthermore, the sensitivity of Mdh isozymes to water temperature has been shown in *F. heteroclitus*, where frequency of the southern allele increased in the warm-water outfall of a nuclear power plant compared to neighboring Long Island populations [46].

#### LDH

One of the initially surprising aspects of our analysis of published data is that the Ldh cline shape is not significantly distinct from neutral expectation, given that much of the functional genetic work pursued by Dennis Powers and colleagues suggested strong diversifying selection [19], [20], [24]. The lack of evidence for diversifying selection when applying fdist2 to Ldh can be explained by looking at the full distribution of clines available (Figure 1, panel B). It is clear that Ldh does not exhibit the steepest slope observed. This gentler slope obviously determines the central location of Ldh in the Co-Co analysis, but it also reduces 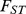 over the full geographic range due to the fact that populations in proximity to the cline location are polymorphic for the two Ldh alleles.

### Analysis of Genomic Clines

In this study we introduce a likelihood-based framework that allows partially unsupervised identification of genetic loci exhibiting clinal patterns of spatial variation. Other analytical methods exist to analyze clinal variation across hybrid zones, though we argue that these methods are not appropriate in the *F. heteroclitus* cline. For example, recent regression-based methods [47] require generation of a hybrid index and are thus inappropriate for geographic clines in which the cline is old, introgression is extensive, or generation of hybrid indices are otherwise impossible. Likewise, models based on primary intergradation [10] are inappropriate for stepped clines generated by secondary contact. Such approaches may become increasingly important in species other than *F. heteroclitus* as more and more large population-genomic datasets are generated through the use of next-generation sequencing techniques.

Both type I and type II error are troublesome when searching for patterns of selection in a population genomic dataset. Type I error, the situation in which a neutral locus is considered to be under selection, can cause a waste of resources as a research team subjects the mis-identified locus to cost- and labor-intensive laboratory methods in an attempt to identify the nature of selection on the molecule. The converse, type II error, where loci under selection are not identified in a population genomic scan, can also waste resources and may also have a more insidious impact.

Use of population genomic scans has been suggested as a means to ensure that all loci in a dataset are behaving neutrally so that they can be used for typical molecular ecological inference [48]; type II error will be positively misleading in this case. Thus, an inability to show that loci are under selection using the distribution of geographic variation may indicate that these loci will not strongly mislead inference on population structure; however, this does not ensure that misidentified loci will not reduce the accuracy of estimates of within-population parameters (for example estimation of family size).

#### Roles of history versus selection in *F. heteroclitus*

Though diversifying selection has been demonstrated for a few loci in *F. heteroclitus* using independent means, demographic history can explain all clinal patterns observed in *F. heteroclitus* without invoking natural selection to either establish or maintain the pattern we observe today. This alternative hypothesis has long been acknowledged but not quantitatively addressed by previous authors [19], [23], [25], [32]. In this study, two lines of evidence support this conclusion. First, in a large sample of loci examined along the cline, nearly half exhibit a significantly clinal pattern. We believe it is unlikely that 44–47% of the loci assayed happened to be under the same diversifying selection pressure. Second, we simulated neutral loci under a likely demographic history of *F. heteroclitus* and found that, compared to empirical data, simulated loci showed similar proportions of loci exhibiting clinal patterns and similar results using both Co-Co and fdist2 approaches.

Though demography can explain allele frequencies, we are asserting only that detecting diversifying selection in this system is difficult, if not impossible, given the strong historical signal. We do not rule out the possibility of diversifying selection in this system, only that geographical analysis is a very low-power technique to detect it.

## Materials and Methods

Sources of genetic data are summarized in Table 1. Allele frequency data for each population were reported for allozyme and mitochondrial loci. Individual-level genotypes for microsatellite and SNP data were converted to allele frequencies before analysis. We removed all SNP loci that were invariant across populations included in this study and also those not assayed in each of the 11 populations surveyed for SNP variation. This reduced our initial set of 310 SNP loci to 285. In order to make analysis consistent across loci, we analyzed all data while assuming Hardy-Weinberg equilibrium within all locus-population combinations and linkage equilibrium among all loci.

### Co-Co Plots

At loci in which more than two alleles were observed, we used an F*_ST_*-based approach to identify a focal allele for clinal analysis. Our aim was to identify the allele with the strongest differentiation among northern and southern portions of the sampled range. We first divided the range of *F. heteroclitus* into northern (north of 42°N), central, and southern populations (south of 34°N). We then estimated F*_ST_* for each multiallelic locus across the northern and southern region while ignoring the central region. For each locus, we chose the allele that contributed most to the total genetic variance observed across populations. Analyses were performed with the R package hierfstat [49].

Once focal alleles were identified, we fit a three-segment “twice-broken-stick” model to the relationship between collection site latitude and focal allele frequency. This model assumes that allele frequencies do not vary with space outside of the cline and that they vary linearly within the cline. Four parameters are fit: the allele frequencies North and South of the cline and the locations of the transitions from non-clinal to clinal portions of the range. These parameters correspond to the coordinates of the “breaks” in latitude/allele frequency space and were estimated using the R function optim. From these four parameters, we calculated the midpoint and slope of each cline.

Modeling clinal variation as three straight line segments represents a significant departure from traditional analyses. It is more common for single-locus clines to be described by a four-parameter logistic model [12] whose parameters describe the frequencies of the focal allele at either end of the geographic transect, the steepness of the cline, and the location of the cline. However, numerical estimation of the logistic model can be difficult when applied in the unsupervised manner required to screen large numbers of loci. In some cases when no clinal pattern exists, failure to converge on the parameter estimates is expected. Unfortunately, in cases in which visual inspection indicates an obvious cline but the difference in allele frequency at the ends of the cline is moderate or the ends of the cline are poorly defined, non-linear regression packages that implement logistic models (SAS; R-package nlme) also fail to converge. Broken-stick models are much less sensitive to these effects. For most loci for which we were able to fit a logistic model, logistic and broken-stick fits were both coincident and concordant (See Figure S1, for example).

We included a given locus in the Co-Co analysis when it passed two criteria. First, the locus showed a significant signal of genetic isolation (i.e., F*_ST_*) by geographic distance, as determined by a Mantel test [50]. Second, a broken-stick fit of the locus represented a significant improvement over the fit of a horizontal line to the data. Comparison between these fits was conducted using likelihood-ratio tests. These criteria identify 125 of an initial 285 SNP loci available (44%) that clinally vary across the broad geographic range of the species. Note that all 285 SNP loci were included in the F*_ST_*-outlier analysis (below).

To identify allelic clines that significantly differed in location or slope, we used a 2-dimensional kernel-density estimated from the coordinates of the cline for each locus in location/slope space. To account for right-skewness in the distribution of cline slopes, we first log-transformed each slope’s absolute value. We then estimated confidence envelopes that encompassed 95, and 99% of the volume of the 2-d kernel density. Bandwidths for 2-dimensional density estimation and creation of envelopes were estimated from the data using cross-validation [51] and were implemented in the R package ks.

### F*_ST_*-based Outlier Analysis

We summarized all raw data into an 82 population × 310 loci matrix of allele frequencies. For subsets of the data in which a particular type of locus (microsatellite, SNP, etc) was not collected for a population, that population was eliminated from analysis for those loci only. We then generated 20 individual-level genotypes at each population-locus combination assuming Hardy-Weinberg Equilibrium and no linkage disequilibrium. These synthetic genotypes were produced using a custom R script (available from authors upon request) and output in GenePop format. This Genepop file was then converted to FDist format using PGDSpider (http://www.cmpg.unibe.ch/software/PGDSpider/) and analyzed using fdist2 [15]. Though alternatives to fdist2 have been developed, we chose this approach because of its frequency of use in the literature and because it has recently been shown to exhibit more reasonable error rates relative to other methods [52]. Because the median number of populations assayed per locus was 11 (range = 7–41), we simulated 100 demes and sampled 11 demes across 50,000 replicate simulations assuming infinite alleles and using F*_ST_* = 0.33 as a starting value. Changing the number of demes to 13 (the mean number of populations assayed per locus) or altering the starting F*_ST_* value did not qualitatively affect the result.

### Simulations

#### Historical biogeography and demography

Our simulations were intended to reflect a historical pattern in which an ancestral population was divided in the late Pleistocene. These two descendants represent relictual, isolated populations. At the start of the Holocene (90,000 generations after isolation), each of these two populations underwent an instantaneous range expansion into 20 descendant populations. The timing of these events are consistent with existing estimates of divergence times along the *Fundulus* cline [35]. All 40 descendant populations were linked to their immediate neighbors through gene flow under a strict stepping-stone model with a constant pairwise rate of exchange, 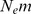, until the present. Figure 3 provides an illustration of the demographic history used in simulations. Total effective population size was held constant throughout the simulation (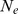 of ancestors is equal to the sum of 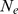
’s of their descendants). We chose an 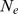 for each population in the present day equal to 2500, based on previously determined estimates of 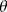 [23] and an assumed microsatellite mutation rate of 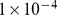.

All simulations were implemented with msms [53]. This software performs coalescent simulation of neutral loci using the same algorithm as ms [54], [55].

## Supporting Information

Figure S1 **Examples of three model fits to clinal data for two loci.** Red corresponds to a logistic fit, green a broken-stick fit, and blue a linear fit. Panel on the left illustrates the similarity between the the logistic and broken-stick models in well-behaved clinal data with large differences in allele frequency between the ends of the cline. Panel on the right corresponds to a situation where a clinal pattern appears in the data, but a logistic fit would not converge using the nlme package available on the R package repository (cran.r-project.org)
(PDF)

Figure S2
**Simulation results for: 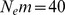, other conditions same as in manuscript.** Symbols and notation employed in plot identical to figure 4 in main document. Note that while the slope of clinally varying loci is less steep, they have the same midpoint and the proportion of loci exhibiting clinal variation is comparable to those cases illustrated in the main document.(PDF)

Table S1 **Loci identified as outliers using Co-Co and fdist2 analyses.**
(PDF)
