# Supplementary figures and images for: Can Diversifying Selection Be Distinguished from History in Geographic Clines? A Population Genomic Study of Killifish (Fundulus heteroclitus)
Source: PLoS One. 2012 Sep 26;7(9):e45138. doi: 10.1371/journal.pone.0045138 (PMC3458873; doi:10.1371/journal.pone.0045138)

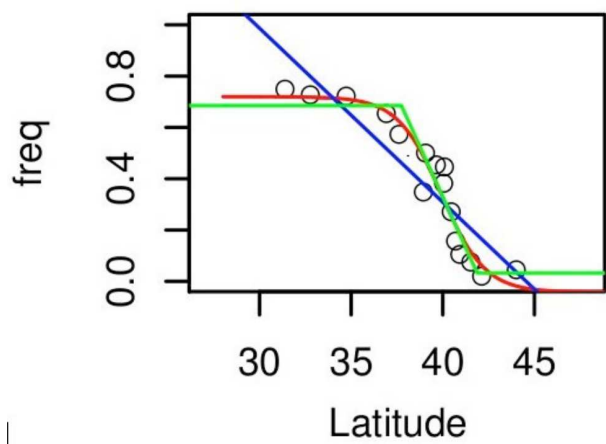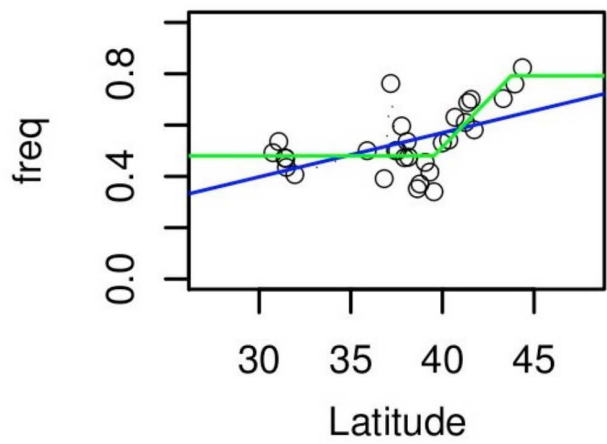

Supplement: Figure S1 — Examples of three model fits to clinal data for two loci. Red corresponds to a logistic fit, green a broken-stick fit, and blue a linear fit. Panel on the left illustrates the similarity between the the logistic and broken-stick models in well-behaved clinal data with large differences in allele frequency between the ends of the cline. Panel on the right corresponds to a situation where a clinal pattern appears in the data, but a logistic fit would not converge using the nlme package available on the R package repository (cran.r-project.org) (PDF) [file pone.0045138.s001.pdf]

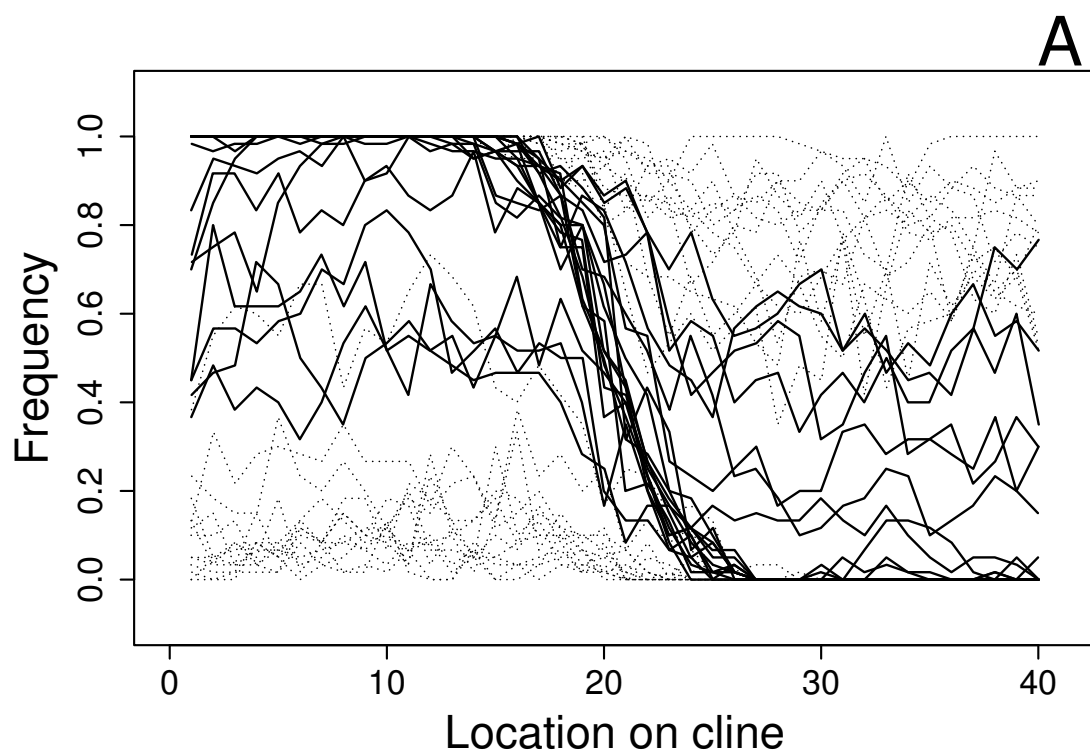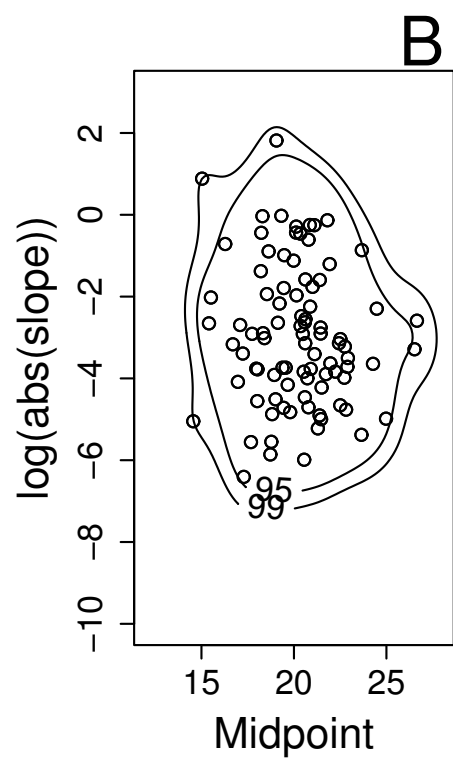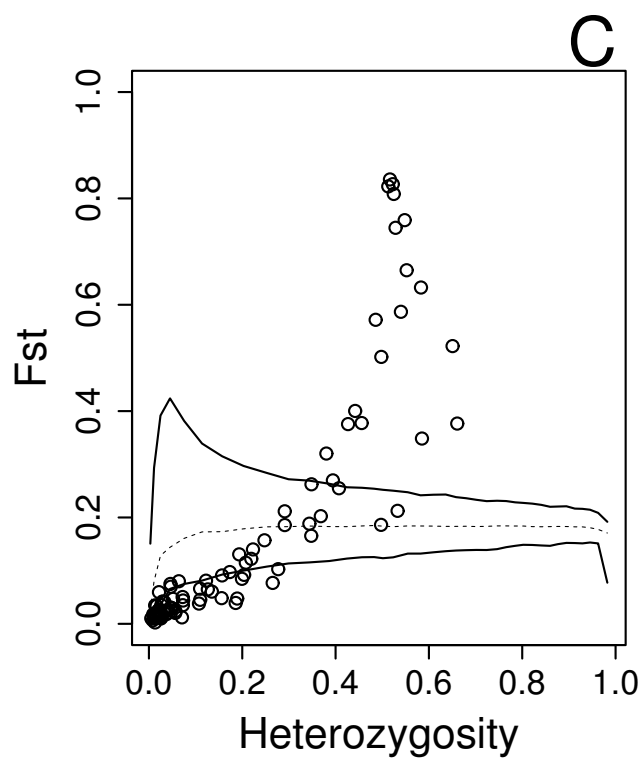

Supplement: Figure S2 — Simulation results for: , other conditions same as in manuscript. Symbols and notation employed in plot identical to figure 4 in main document. Note that while the slope of clinally varying loci is less steep, they have the same midpoint and the proportion of loci exhibiting clinal variation is comparable to those cases illustrated in the main document. (PDF) [file pone.0045138.s002.pdf]
