# Supplementary material for: Can Diversifying Selection Be Distinguished from History in Geographic Clines? A Population Genomic Study of Killifish (Fundulus heteroclitus)
Source: PLoS One. 2012 Sep 26;7(9):e45138. doi: 10.1371/journal.pone.0045138 (PMC3458873; doi:10.1371/journal.pone.0045138)
Supplement: Table S1 — Loci identified as outliers using Co-Co and fdist2 analyses. (PDF) [file pone.0045138.s003.pdf]

Table S 1: Loci identified as outliers using Co-Co and `fdist2` analyses

| Locus | Type | CoCo | fdist2 |
|---|---|---|---|
| x280_83 | SNP | | X |
| x65_135 | SNP | | X |
| xCONTIG_538_2_COX1_SQUAC_CYTO_ | SNP | | X |
| xTC15227_467 | SNP | | X |
| xTC15297_568 | SNP | | X |
| xTC15306_666 | SNP | | X |
| xTC15344_885 | SNP | | X |
| xTC15361_229 | SNP | | X |
| xTC15412_571 | SNP | | X |
| xTC15413_237 | SNP | | X |
| xTC15431_171 | SNP | | X |
| xTC15477_258 | SNP | | X |
| xTC15759_647 | SNP | | X |
| xTC15866_633 | SNP | | X |
| xTC15936_105 | SNP | | X |
| xTC16027_529 | SNP | | X |
| xTC16344_233 | SNP | | X |
| xTC16441_544 | SNP | | X |
| xTC17468_80 | SNP | | X |
| xTC17785_524 | SNP | | X |
| xTC18194_242 | SNP | | X |
| xTC18652_242 | SNP | | X |
| xTC18885_458 | SNP | | X |
| xTC19007_297 | SNP | | X |
| xTC22196_102 | SNP | | X |
| mtdna | mtDNA | X | X |
| mdh | Allozyme | X | X |
| xCONTIG_966_MDMC_STRMY.1 | SNP | X | |
| x1328_135.1 | SNP | X | |
| xTC17598_212.1 | SNP | X | |
